# In-Hospital Postoperative Mortality Rates for Selected Procedures in Tanzania’s Lake Zone

**DOI:** 10.1007/s00268-020-05802-w

**Published:** 2020-09-29

**Authors:** Taylor Wurdeman, Christopher Strader, Shehnaz Alidina, David Barash, Isabelle Citron, Ntuli Kapologwe, Erastus Maina, Fabian Massaga, Adelina Mazhiqi, John G. Meara, Gopal Menon, Cheri Reynolds, Meaghan Sydlowski, John Varallo, Sarah Maongezi, Mpoki Ulisubisya

**Affiliations:** 1 Program in Global Surgery and Social Change, Harvard Medical School, 641 Huntington Avenue, Boston, MA 02115 USA; 2 University of Miami Miller School of Medicine, Miami, FL USA; 3 Department of Surgery, University of Massachusetts, Worcester, MA USA; 4 GE Foundation, Boston, MA USA; 5 Department of Health, Social Welfare and Nutrition Service, President’s Office – Regional Administration and Local Government, Dodoma, Tanzania; 6 Dalberg Advisors, New York, NY USA; 7 Bugando Medical Centre, Consultant and Teaching University Hospital, Mwanza, Tanzania; 8 Department of Internal Medicine, Ängelholm Hospital, Ängelholm, Sweden; 9 Department of Plastic and Oral Surgery, Boston Children’s Hospital, Boston, MA USA; 10 Assist International, Ripon, CA USA; 11 Jhpiego, Baltimore, MD USA; 12 Ministry of Health, Community Development, Gender, Elderly & Children, Dodoma, Tanzania

## Abstract

**Background:**

Postoperative mortality rate is one of six surgical indicators identified by the Lancet Commission on Global Surgery for monitoring access to high-quality surgical care. The primary aim of this study was to measure the postoperative mortality rate in Tanzania’s Lake Zone to provide a baseline for surgical strengthening efforts. The secondary aim was to measure the effect of Safe Surgery 2020, a multi-component intervention to improve surgical quality, on postoperative mortality after 10 months.

**Methods:**

We prospectively collected data on postoperative mortality from 20 health centers, district hospitals, and regional hospitals in Tanzania’s Lake Zone over two time periods: pre-intervention (February to April 2018) and post-intervention (March to May 2019). We analyzed postoperative mortality rates by procedure type. We used logistic regression to determine the impact of Safe Surgery 2020 on postoperative mortality.

**Results:**

The overall average in-hospital non-obstetric postoperative mortality rate for all surgery procedures was 2.62%. The postoperative mortality rates for laparotomy were 3.92% and for cesarean delivery was 0.24%. Logistic regression demonstrated no difference in the postoperative mortality rate after the Safe Surgery 2020 intervention.

**Conclusions:**

Our results inform national surgical planning in Tanzania by providing a sub-national baseline estimate of postoperative mortality rates for multiple surgical procedures and serve as a basis from which to measure the impact of future surgical quality interventions. Our study showed no improvement in postoperative mortality after implementation of Safe Surgery 2020, possibly due to low power to detect change.

**Electronic supplementary material:**

The online version of this article (10.1007/s00268-020-05802-w) contains supplementary material, which is available to authorized users.

## Introduction

Surgery is an integral part of effective health systems, with surgical disease accounting for 30% of the global burden of disease [1]. The Lancet Commission on Global Surgery (LCoGS) identified that 5 billion people lack access to safe, timely, affordable surgical and anesthesia care [2]. Low- and middle-income countries (LMICs) bear the majority of the burden of surgical disease [3]. If surgical care was scaled up in LMICs, 1.4 million deaths could be averted annually [3]. Scaling surgical care in LMICs requires a focus on maternal surgical care, as cesarean section is one of the most common surgical procedures in LMICs [4] and is a necessity for maternal/neonatal survival in 19% of all deliveries [5]. LMICs need to perform 143 million additional surgical procedures per year in order to match the surgical burden of disease [2]. Reaching these goals requires augmentation of surgical provider workforce, scalable technology, improved data quality, and financial access to surgery.

While scaling surgical capacity is important, efforts must also address surgical quality. The postoperative mortality rate (POMR) of elective surgeries in Africa is twice the global average [6]. Furthermore, a recent study found that African women are 50 times more likely than non-African women to die following a cesarean delivery [7]. Surgical and anesthesia systems without quality and safety lead to increased rates of surgical complications, including death [8–10]. While quality is difficult to codify, POMR has been identified as an important indicator encompassing multiple surgical complications. POMR is one of six surgical indicators identified by LCoGS for monitoring access to high-quality surgical care [2]. POMR highlights systems with poor surgical quality or late disease presentation due to poor access to care and gives a starting point to measure precipitating factors. While POMR has been identified as an important indicator, difficulties in measurement have led to few reliable national estimates. The 2016 report on Surgical World Development Indicators showed only 29 countries reporting on perioperative mortality [11]. Historically, POMR has been difficult to track consistently in LMICs, partly due to lack of robust health information systems in many LMICs. Very few studies in Africa report POMR [6, 12]. One difficulty in generating national POMR estimates is the heterogeneity of definitions. The World Health Organization (WHO) and the LCoGS define POMR as death before discharge from the hospital or within 30 days, whichever is sooner [13]. Despite the agreed definition, some studies have included deaths outside of the study hospital. Crude measurements of POMR include every surgical procedure in the denominator, making them sensitive to differences in complexity of cases between hospitals. Procedure-specific POMR may provide a more valid quality measure by capturing in the denominator only cases where there is a true risk of death. This study defines POMR as deaths before discharge or within 30 days, starting the moment the patient leaves the operating room.

Baseline measurement of POMR, while influenced by future changes in case numbers or case mix, is nonetheless a valid measure to monitor changes in quality over time. This paper combines prospective data collection with data from hospital medical records to estimate inpatient POMR for multiple procedure categories in 20 facilities in Tanzania’s Lake Zone. Our primary aim of this study is to provide baseline measurements of POMR in Tanzania’s Lake Zone. Our secondary aim is to evaluate the effect of the Safe Surgery 2020 (SS2020) intervention on POMR after one year.

## Materials and methods

### Study design

This prospective, longitudinal study of POMR was designed to provide a baseline measurement of POMR in Tanzania’s Lake Zone and to assess the impact of the SS2020 intervention on in-hospital POMR.

### Study setting and participants

**Fig. 1:**
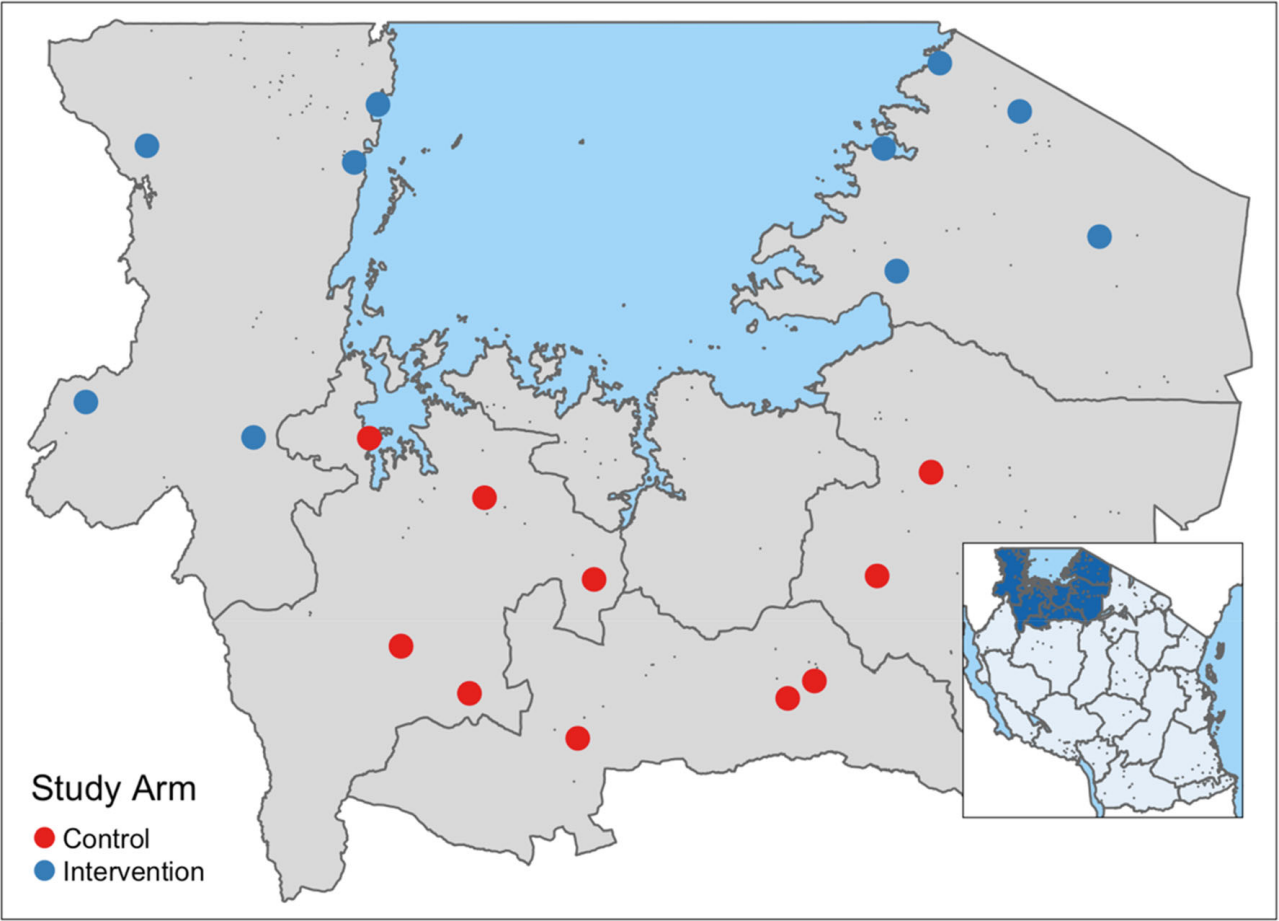
Map of study sites in the Lake Zone of Tanzania

This study took place at 20 facilities in Tanzania’s Lake Zone including health centers, district hospitals, and regional hospitals (Table 1). The facilities were divided into two groups based on their geographical location (Fig. 1). Surgical inpatients including cesarean delivery were included. Vaginal deliveries were also followed to provide comparison data.

**Table 1:** Facility characteristics are presented to show similarity of pre-intervention characteristics between control and intervention sites

Characteristics	All facilities (*n* = 20) *n* (%)	Intervention facilities (*n* = 10) *n* (%)	Control facilities (*n* = 10) *n* (%)
*Level of facility*			
Health centre	4 (20%)	2 (20%)	2 (20%)
District hospitals	11(55%)	6 (60%)	5 (50%)
Regional referral hospital	5 (25%)	2 (20%)	3 (30%)
*Number of inpatient beds*			
0–100	5 (25%)	3 (30%)	2 (20%)
101–300	13 (65%)	6 (60%)	7 (70%)
300+	2 (10%)	1 (10%)	1 (10%)
Average number of major surgeries per facility	102	52	50
Number of functioning major ORs per facility	1.7	1.6	1.7
Average monthly inpatient volume per facility	589	330	258

Multiple hospital classifications and sizes were used for this study

### Intervention

The SS2020 multicomponent intervention was implemented over 10 months to improve surgical quality. The first phase involved training surgical team members on leadership, teamwork, communication, and quality improvement techniques. The second phase involved training on the WHO’s Surgical Safety Checklist (SSC), peri-operative infection prevention, safe cesarean delivery, sterilization protocols, data quality, and surgical techniques. The third phase involved bimonthly mentorship visits, virtual mentorship through Project ECHO, infrastructure grants up to $10,000 USD, an equipment package, and the Touch Surgery mobile training application. A more detailed description of the intervention is described elsewhere [14].

### Data collection

Tanzanian medical doctors were trained to collect data for the study, with each facility having at least one dedicated full-time data collector. Data were collected over two time periods: pre-intervention (February–April 2018) and post-intervention (March–May 2019). All elective and emergency surgeries were observed on weekdays, and all emergencies at the weekends. Patients were evaluated starting postoperative day one for surgical procedures and from day zero for spontaneous vaginal deliveries. Data collectors made daily rounds to each surgical and postpartum inpatients and for up to 30 days or discharge, documenting the patient’s mortality status. For this study, data were collected on demographics, surgery type, and time until death, where applicable. All data were collected using paper forms and transferred to REDCap daily.

### Analysis

We define postoperative mortality as any death following a surgical or obstetric procedure within 30 days of the procedure or until discharge, whichever is sooner. Surgery patients discharged before 30 days who subsequently died at home or another hospital were not followed. Intra-operative deaths were also not included due to limitations in data collector availability, with observation of patients starting when they arrived to the postoperative ward. The denominator for the POMR calculation was the number of major surgeries, defined as a procedure requiring general/regional anesthesia. POMR was stratified based on surgical category. Due to the relatively small number of deaths, three categories were used: cesarean delivery, laparotomy, and surgeries (excluding laparotomy) (see Table 2 footnotes for a full list). The definition of laparotomy for this study was a midline incision of the abdomen of varying lengths, which is more broad as compared with the Western definition of laparotomy. Examples in our data collection included classical laparotomy, total abdominal hysterectomy, and splenectomy. Procedures listed as laparotomy, but involving a horizontal incision as determined by clinical experience of an HIC surgeon, were excluded. To analyze the effect of the intervention on postoperative mortality, a logistic regression model was used with an interaction term between intervention status and time point. Unless explicitly stated, all results are presented as aggregates across control and intervention sites.

**Table 2:** Aggregated postoperative mortality rate by procedure type

Cohort	Number of deaths	Number of procedures	Postoperative/CS mortality rate	Mean length of stay in days, dead (sd)	Mean length of stay, alive (sd)
Only laparotomies	30	765	3.92%	5.1 (6.3)	5.3 (4.3)
All surgeries, excluding laparotomies^a^	12	841	1.43%	3.5 (3.8)	3.9 (3.3)
Only cesarean deliveries	11	4505	0.24%	2.5 (2.2)	3.8 (3.1)

The data is aggregated across study arms

^a^This category includes hernia repair (4 deaths), prostatectomy (3 deaths), amputation (2 deaths), debridement (1 death), disarticulation (1 death), and hydrocelectomy (1 death)

### Power calculation

The study is predicted to be powered to 0.33–0.72 for changes in non-obstetric POMR and 0.22 for cesarean delivery specific POMR before and after the SS2020 intervention. To adequately power the question of whether or not the SS2020 intervention had an effect on POMR would have required 797 non-obstetric surgeries and 7985 cesarean deliveries in the pre-intervention and post-intervention arms. Reaching these case numbers was not feasible within the larger SS2020 effort due to time and resource constraints. A full evaluation protocol of the SS2020 project can be read in Alidina et al. 2019 [14]. The details of assumptions for the power calculations can be found in Online Resource 1.

## Results

The mean age of patients was 29.3 years. Females comprised 89.7% of the study population. The mean age of patients who required non-obstetric surgical procedures was 40.1 years. Table 3 describes the patient characteristics. Laparotomies accounted for 48.0% of non-obstetric procedures. Bellwether procedures, defined by LCoGS as the three procedures (cesarean delivery, laparotomy, and treatment of open fracture) that any well-equipped surgical center should provide, made up 86.2% of procedures. The majority (85.4%) of the bellwether procedures conducted were cesarean deliveries.

**Table 3:** Demographic information and procedure volume for each study arm

	Pre-intervention		Post-intervention	
	Control	Intervention	Control	Intervention
*Non-obstetric surgeries*				
Age, mean (sd)	41.2 (18.4)	38.6 (18)	42.3 (19.7)	38.2 (19.4)
Percent female	268/431 (62.2%)	260/412 (63.1%)	231/413 (55.9%)	232/382 (60.7%)
# Laparotomies/total non-obstetric procedures	227/431 (52.7%)	179/412 (43.4%)	202/413 (48.9%)	179/382 (46.9%)
# Other procedures/total non-obstetric procedures	204/431 (47.3%)	233/412 (56.6%)	211/413 (51.1%)	203/382 (53.1%)
*Cesarean delivery*				
Age obstetric, mean (sd)	25.4 (6.3)	24.9 (6.2)	25.9 (6.5)	25.5 (6.5)
# Cesarean deliveries/total procedures (%)	1112/1536^a^ (72.4%)	1123/1535^a^ (73.2%)	1089/1499^a^ (72.6%)	1208/1588^a^ (76.1%)

^a^Denominator represents the total number of surgeries, including cesarean delivery and all other surgeries. Twelve procedures were classified into multiple categories. For example, in some cesarean deliveries, a laparotomy was also performed

Among the 6,158 patients followed across the intervention and control sites, there were 53 deaths. The in-hospital non-obstetric POMR was 2.62%. The laparotomy specific POMR was 3.92%. Cesarean delivery POMR was lower than non-obstetric surgery POMR at 0.24%. Cesarean delivery mortality was 8 times higher than spontaneous vaginal delivery mortality (0.24% vs. 0.03%). All of the deaths occurred during emergent cesarean deliveries. Table 2 presents the POMR and average length of stay for each procedure category.

Laparotomy POMR ranged between 3.37 and 4.52% among the study cohorts. The non-obstetric, non-laparotomy POMR ranged between 0.99 and 2.44%. Cesarean delivery mortality ranged between 0.17 and 0.36%. Logistic regression demonstrated no statistically significant effect of the SS2020 intervention on POMR in any of the surgical procedure categories (Table 4). POMR rates were not risk adjusted due to missing data. However, urgency, ASA score, and wound class by procedure category are presented in Table 5 for reference.

**Table 4:** Postoperative mortality rates for each study arm and procedure category

	Pre-intervention		Post-intervention		*p*-value
	Control (%)	Intervention (%)	Control (%)	Intervention (%)	
*Non-obstetric surgeries*					
Only laparotomies	4	3.37	4.06	4.52	0.701
All surgeries, excluding laparotomies/cesarean delivery	0.99	1.30	2.44	0.99	0.283
*Obstetric*					
Cesarean delivery	0.27	0.36	0.19	0.17	0.718

**Table 5:** Risk factor-specific mortality rates

Surgery group	Risk factor		Mortality rate	% Missing data^a^
Laparotomy	Urgency	Elective	0.57% (1/174)	39.1%
		Emergent	3.77% (11/292)	
	ASA	ASA 1	2.13% (4/188)	45.1%
		ASA 2	4.31% (9/209)	
		ASA 3	0% (0/20)	
		ASA 4	0% (0/1)	
		ASA 5	0% (0/2)	
	Wound class	Clean	3.06% (9/294)	35.8%
		Clean-Contaminated	0.76% (1/132)	
		Contaminated	4.35% (2/46)	
		Dirty	5.26% (1/19)	
Surgeries (excluding laparotomy)	Urgency	Elective	2.03% (7/345)	41.7%
		Emergent	2.07% (3/145)	
	ASA	ASA 1	0.9% (2/223)	52.7%
		ASA 2	3.14% (5/159)	
		ASA 3	0% (0/7)	
		ASA 4	0% (0/1)	
		ASA 5	0% (0/8)	
	Wound class	Clean	1.47% (6/408)	37.8%
		Clean-Contaminated	1.25% (1/80)	
		Contaminated	5% (1/20)	
		Dirty	6.67% (1/15)	
Cesarean delivery	Urgency	Elective	0% (0/203)	32.9%
		Emergent	0.28% (8/2819)	
	ASA	ASA 1	N/A	38.4%
		ASA 2	0.29% (8/2724)	
		ASA 3	0% (0/21)	
		ASA 4	0% (0/5)	
		ASA 5	0% (0/23)	
	Wound class	Clean	N/A	30.9%
		Clean-Contaminated	0.26% (8/3077)	
		Contaminated	11.54% (3/26)	
		Dirty	0% (0/11)	

Risk factor data were taken from the OR logbook and matched to the surveillance tool for mortality. No risk adjustment was completed due to missing data

^a^Urgency, ASA, and wound class were recorded using the OR logbook, which is not directly linked to the surveillance tool for mortality. The two tools were combined using demographic data, procedure date, and location. Between 30.9 and 52.7% of data were missing due to incompatibility of tools

## Discussion

This study provides POMR estimates for various procedure categories in Tanzania’s Lake Zone. The POMR for laparotomy (3.92%) was highest compared to non-laparotomy POMR (1.43%) and cesarean delivery POMR (0.24%). This relative difference is likely due to the risk profile associated with laparotomies, but also highlights issues such as late presentation of disease. Despite the predictably higher rate of mortality of laparotomies compared to other surgeries in this study, it is lower than laparotomy estimates in other studies. A systematic review of LMICs showed a median POMR of 11.11% in laparotomies [15]. A study on laparotomy for gastric outlet obstruction in Tanzania found a 18.5% mortality rate [16]. The disparity with our laparotomy POMR estimates may be attributable to a relatively young population, referral of complex patients to tertiary referral hospitals, surgeon confidence in complex cases, or deaths occurring before presenting to the hospital. For example, the Million Death Study in India found that 71% of deaths due to acute abdominal conditions occurred at home [17]. Different health practices, cultural norms, healthcare referral structure, and infrastructure can contribute to death prior to arrival. The relatively high rate of clean wound class laparotomies in this study suggests that sicker patients may be dying before arrival or referred onto higher levels of care.

Cesarean delivery mortality (0.24%) was significantly higher than the maternal mortality associated with spontaneous vaginal deliveries (SVD) (0.03%). Patients who had cesarean delivery on average stayed longer in the hospital and had more complicated deliveries. Many SVD patients were discharged quickly, potentially resulting in underreported deaths. A higher POMR from cesarean delivery compared to SVD is consistent with international literature [18]. Compared to data from the ASOS study [6], the cesarean delivery mortality in this study was 2.2 times lower (0.24% vs. 0.53%). In another study, it was 5.5 times lower when compared to other low-income countries (0.24% vs. 1.32%) and 30 times higher when compared to high-income countries like the United Kingdom (0.24% vs. 0.008%) [19]. The mortality from the current study is underestimated compared to other low-income estimates, as it did not capture intraoperative death, a common time of death in severe postpartum hemorrhage. The cesarean delivery mortality in this study more appropriately approximates emergent mortality, as there were zero deaths among elective cases. Transition to a higher proportion of elective cesarean deliveries, as is seen in high-income countries, could decrease mortality rates.

Patients died early in their course of stay, with 83% of patients dying in the first 7 days. This finding suggests that the cause of high mortality may reflect preoperative, intraoperative, or early postoperative factors. Preoperative factors include patient comorbidities, late presentation for surgery, and presurgical vital status. This finding is consistent with the ASOS study, in which 94.1% of mortalities occurred on day one [6]. With the majority of deaths occurring early during admission, the case can be made for reducing the follow-up time for POMR. While this has been shown to lead to underestimation of 30-day POMR [20], a shorter follow-up period would be more feasible for data monitoring in LMICs [21].

After 10 months of the SS2020 intervention, we did not observe a statistically significant effect on POMR. The study was underpowered to detect a change in pre–post-intervention POMR between the control and intervention groups. Therefore, no conclusions can be drawn on the effectiveness of the intervention on reducing POMR. A larger sample size and a longer intervention period may detect an effect of the SS2020 intervention on POMR. Similar studies implementing the surgical safety checklist alone have demonstrated a significant reduction in POMR after just one year [22, 23]. One intervention in Tanzania reduced POMR from 5.67 to 2.93% after implementation of a “Continuous Quality Improvement” approach including preoperative visits the day before surgery and appropriate medical management of patients after surgery [12]. The study had an intervention follow-up period of 1 and 2 years and a large sample contributing to their ability to detect changes.

Surgical care is becoming increasingly prioritized in global health efforts, as evident in the development of the National Surgical, Obstetric, and Anesthesia Plan (NSOAP) in Tanzania in 2018 [24] and the Southern African Development Community (SADC) [25] resolution calling for all SADC countries to create NSOAPs. NSOAP’s are a response to the recognition that access to surgical care is a public health issue. They contain recommendations on monitoring surgical indicators to assess the current state of surgical capacity and monitor progress towards capacity building. This study provides data on POMR as one key surgical indicator required in the Tanzania NSOAP and can serve as a baseline for tracking surgical quality. The lack of power in this study highlights the need for large national datasets to benchmark and track changes in POMR. As surgery in LMICs are scaled, the quality of surgical care must be continuously monitored due to the potential for increasing POMR with increasing surgical volume [26]. This focus on surgical system quality alongside scaling of surgical delivery is essential for the eventual transformation of LMIC surgical systems [27].

## Limitations

The number of deaths in this study is likely an underestimate as discharged or transferred patients were not followed after their hospital stay. Furthermore, as patients were only observed after surgery, deaths during surgery are also missing. Intra-operative deaths are an important measure of anesthesia safety and should be included in future investigations. This study focused on a small number of Tanzanian health facilities, with interventions implemented at only 10 facilities. The lack of power to detect changes in POMR resulting from the SS2020 intervention at control and intervention sites was anticipated, which is why a diverse array of indicators were used to evaluate the impact of the intervention. Future studies should consider larger samples to ensure that it is powered to detect changes in POMR.

## Conclusions

This study provides estimates of in-hospital POMR for multiple procedure categories in the SS2020 study population in the Lake Zone of Tanzania. These results should be used as a baseline for measurement of future POMR as the Tanzania NSOAP continues to be implemented and evaluated. This study also showed no improvement in POMR immediately after implementation of the SS2020 intervention, possibly due to low power to detect change.

## Electronic supplementary material

Below is the link to the electronic supplementary material.

Supplementary file1 (PDF 65 kb)
