# Supplementary material for: In-Hospital Postoperative Mortality Rates for Selected Procedures in Tanzania’s Lake Zone
Source: World J Surg. 2020 Sep 29;45(1):41–9. doi: 10.1007/s00268-020-05802-w (PMC7752880; doi:10.1007/s00268-020-05802-w)
Supplement: Supplementary file 1 — Supplementary file1 (PDF 65 kb) [file 268_2020_5802_MOESM1_ESM.pdf]

Supplementary Appendix

Manuscript title: In-hospital post-operative mortality rates for selected procedures in Tanzania's Lake Zone

Online Resource 1: Power Calculation

The power calculation for this study requires a few assumptions. Given an estimated average surgical volume of 75 procedures per intervention facility per month (Alidina et al 2019), there would be 2250 procedures in the pre-intervention cohort and 2250 procedures in the post-intervention cohort. An estimated 80-90% of those procedures are caesarian deliveries. For the purposes of the power calculation, 85% of procedures equals 1913 caesarean deliveries per cohort. The remaining 15% of procedures are non-obstetric procedures, equaling 338 procedures. Estimates of non-obstetric POMR in this region vary widely by source, from 5.67% in a prospective study (Bosse et al 2015) to 11.11% in a systematic review (Ng-Kamstra et al 2018). Because the baseline rate is unknown prior to the study, both of these extremes will be used to calculate power. Estimates of caesarian delivery mortality in the region are 0.61% (Biccard et al 2018). A 50% reduction of POMR and caesarian delivery mortality will be used as a clinically significant endpoint for power calculation. As the procedure categories used in this study are not widely used in existing literature to report POMR, power is only calculated for these two categories. Fishers exact test is used for power calculation. The specific power calculations are included below.

Type of Procedure	# of procedures (pre-intervention, post-intervention)	Pre-intervention mortality rate	Targeted post-intervention mortality rate	Power
Non-obstetric	338, 338	5.67%	2.84%	0.33
Non-obstetric	338, 338	11.11%	5.56%	0.72
Caesarian Delivery	1913	0.61%	0.31%	0.22

Per the power calculations, this study is underpowered to detect a true difference in mortality. This was known prior to the start of the study, given the generally low incidence of mortality after surgery. For this reason, POMR was considered a secondary outcome of the larger SS2020 project, with more proximal outcomes (for example, SSC adherence) being used to determine intervention success.

This study resulted in a lower baseline non-obstetric POMR than expected, further reducing the power to detect true differences in POMR. Similarly, the baseline caesarian delivery mortality was lower than expected.
